# Dural administration of inflammatory soup or Complete Freund’s Adjuvant induces activation and inflammatory response in the rat trigeminal ganglion

**DOI:** 10.1186/s10194-015-0564-y

**Published:** 2015-09-02

**Authors:** M. Lukács, KA Haanes, Zs. Majláth, J. Tajti, L. Vécsei, K. Warfvinge, L. Edvinsson

**Affiliations:** Department of Medicine, Institute of Clinical Sciences, Division of Experimental Vascular Research, Lund University, Sölvegatan 17, SE 221 84 Lund, Sweden; Department of Clinical Experimental Research, Copenhagen University, Glostrup Hospital, Copenhagen, Denmark; Department of Neurology, University of Szeged, Szeged, Hungary; MTA-SZTE Neuroscience Research Group, Szeged, Hungary

**Keywords:** Inflammatory soup, Complete Freund’s Adjuvant, Dura mater, Trigeminal ganglion, pERK1/2, IL-1β, CGRP

## Abstract

**Background:**

Migraine is a painful disorder with a huge impact on individual and public health. We hypothesize that migraine pain originates from a central mechanism that results secondarily in hypersensitivity in peripheral afferents associated with the cerebral and cranial blood vessels. It has previously been shown that application of inflammatory or algesic substances onto the dura mater or chemical stimulation of the dural receptive fields causes hypersensitivity to mechanical and thermal stimulation together with direct activation of the TG. We asked whether local inflammation of dura mater induces inflammatory activation in the trigeminal ganglion.

**Methods:**

We performed topical administration of inflammatory soup (IS) or Complete Freund’s Adjuvant (CFA) onto an exposed area of the rat dura mater *in vivo* for 20 min. The window was closed and the rats were sacrificed after 4 h and up to 7 days. Myography was performed on middle meningeal arteries. The trigeminal ganglia were removed and processed for immunohistochemistry or Western blot.

**Results:**

Both CFA and IS induced enhanced expression of pERK1/2, IL-1β and CGRP in the trigeminal ganglia. The pERK1/2 immunoreactivity was mainly seen in the satellite glial cells, while IL-1β reactivity was observed in the neuronal cytoplasm, close to the cell membrane, seemingly as sign of neuro-glial interaction. The CGRP expression in the neurons and nerve fibres was enhanced after the application of either inflammatory agent. Myography resulted in a strong vasoconstrictor response to IS, but not to CFA.

**Conclusions:**

These results suggest that the application of IS or CFA onto the dura mater causes long-term activation of the TG and demonstrate the importance of the neuro-glial interaction in the activation of the trigeminovascular system.

**Electronic supplementary material:**

The online version of this article (doi:10.1186/s10194-015-0564-y) contains supplementary material, which is available to authorized users.

## Background

Migraine is a painful, debilitating neurological disorder with a huge impact on individual and public health. In a survey about years lived with disability, migraine was ranked third [1].

Great progress has been made towards understanding the pathophysiology of migraine; however some questions still remain regarding the origin of migrainous pain and its chronification. There are two contrasting theories of migraine pathogenesis: the neurogenic theory suggests that migraine is a CNS disorder involving a genetic change in the central ion channels, with underlying importance of the phenomenon of cortical spreading depression (CSD). This may lead to activation of the nociceptive afferents causing alteration in the diameter of intracranial vessels, serving as a trigger or modifier in the trigeminovascular system [2]. On the other hand, Ray and Wolff in the 40’s put forward the vasogenic theory, suggesting that migraine is a vascular disorder, caused by a short period of vasoconstriction and a reactive vasodilatation of the cranial arteries (2–5). We hypothesize that migraine pain originates from a central mechanism that secondarily results in hypersensitivity in peripheral afferents associated with the cerebral and cranial blood vessels, thus underscoring the crucial role of trigeminal perivascular nociceptive afferents [3, 4]. The dural afferents might be activated further during dural exposure to inflammatory substances or when the dural mast cells are degranulated [5].

The trigeminal ganglion (TG) with its bipolar neurons, surrounded by satellite glial cells (SGCs), forms the converging point between the CNS and peripheral structures such as the cranial arteries. We are interested in whether local inflammation can induce alterations in the TG neurons and SGCs. Previous experiments involving the use of cell culture [6] or TG organ culture [7] demonstrate that activation of intracellular protein kinases could be rapidly induced with an inflammatory response within the TG. It has also been shown that application of inflammatory or algesic substances onto the dura mater or chemical stimulation of the dural receptive fields causes hypersensitivity to mechanical and thermal stimulation and also activation in the TG and in the brainstem trigeminal neurons [8–11]. We consider that this may serve as a suitable method for *in vivo* examination. In support of this view, infusion of nitroglycerine or local capsaicin onto the dura mater elicits increased extracellular signal-regulated kinase (ERK) phosphorylation in the meningeal arteries and TG [12, 13].

An important component in the inflammatory response is IL-1β, which has been shown to be implicated in neuropathic pain. Overall previous findings reveal the importance and complexity of interaction between neurons, glial and immune cells, playing an important role in the maintenance of neuropathic pain after nerve injuries [14–16].

Neuroscientists ascribe major importance to calcitonin gene-related peptide (CGRP) in the pathophysiology of migraine, in view of its vasodilatory effect and role in the transmission of nociception. CGRP receptor antagonist could offer an approach in the anti-migraine therapy [17, 18]. *In vitro* studies using inflammatory cocktails (histamine, bradykinin, serotonin, prostaglandin E2) on cultured trigeminal neurons have shown large increase of CGRP release [19].

The aim of the present study was to observe the activation of rat TG neurons and SGCs *in vivo* after dural application of inflammatory soup (IS) or Complete Freund’s Adjuvant (CFA).

## Methods

### Animal procedures

Adult male Sprague–Dawley rats (220–320 g) were used (*n* = 95, 48 for immunohistochemistry, 42 for Western blot and 5 for myography). The animals were raised and maintained under standard laboratory conditions. The study followed the guidelines of the European Communities Council (86/609/ECC) and was approved by the Committee of the Animal Research of University of Szeged (I-74-12/2012) and the Scientific Ethics Committee for Animal Research of the Protection of Animals Advisory Board (XI./352/2012).

Prior to interventions, the animals were deeply anesthetized with an intraperitoneal injection of 4 % chloral hydrate (0.01 ml/g body weight, Sigma-Aldrich, St. Louis, MO, USA). The head of the animal was fixated in a stereotaxic frame and a handheld drill was used to remove a 3x3 mm portion of the skull. The hole was made postero-laterally to the bregma (5 mm), on the left side, care being taken not to penetrate the dura mater.

In 18 animals IS was applied onto the dural surface, in 18 animals CFA (inactivated and dried Mycobacterium tuberculosis in mineral oil; Sigma-Aldrich, St. Louis, MO, USA) and in 9 animals (controls) physiological saline was applied as vehicle. As absolute controls, three unoperated rats (fresh) were used. IS contained 10 μM bradykinin, 10 μM serotonin,10 μM prostaglandin E2 and 100 μM histamine, pH 5.0; the recipe was from Strassman et al. [9]. Ten μl of IS, CFA or vehicle was applied, and left on the dural surface for 20 min. To prevent the substances from spreading, the head of the animal was adjusted in the stereotaxic frame so that the dural surface covered with the liquid was completely horizontal. After 20 min, the IS or CFA was washed out with saline, bone wax was used to cover the hole and the wound was sutured with three stitches. The effect of IS, CFA and vehicle were examined after 4 h, 24 h and 7 days. The animals did not receive any analgesic after the surgery. At the end-points the animals were fixation-perfused transcardially with 4 % paraformaldehyde in 0.1 M phosphate-buffer (PB) for immunohistochemistry. TG was removed from both sides. After post-fixation for one day in 4 % paraformaldehyde in 0.1 M phosphate buffer (PB), the specimens were cryoprotected using 10 %, 20 % and 30 % glucose in 0.1 M PB. Specimens were frozen on dry ice, stored at −80 °C and subsequently embedded in gelatine medium (30 % egg albumin, 3 % gelatin), cryosectioned at 12 μm and stored at −20 °C until use.

### Hematoxylin-Eosin (HE) staining and immunohistochemistry

Sections were HE stained, using standard protocol (Htx 4 min, Eosin 30 s) for orientation and examination of tissue conditions.

Immunofluorescence staining was performed to demonstrate the localisation of pERK1/2, IL-1β and CGRP in the TG. Sections were thawed at room temperature, then rehydrated in phosphate buffer saline (PBS) containing 0.25 % Triton X-100 (PBS-T) for 15 min. Sections were exposed to primary antibodies in PBS-T containing 1 % bovine serum albumin (BSA) and incubated overnight at 4 °C. After incubation with the primary antibody (see Table 1), sections were equilibrated at room temperature, rinsed in PBS-T for 3x15 min, followed by incubation with the secondary antibody for 1 h in a dark room, at room temperature (see Table 2). Sections were washed with PBS-T for 3x15 min and mounted with anti-fading mounting medium (Vectashield; Vector Laboratories, Burlingame, CA, USA). For nucleus staining Vectashield medium containing 4,6- diamino-2-phenylindole (DAPI; Vector Laboratories) was used. Samples in which the primary antibody was omitted served as negative controls. Double immunohistochemistry with primary antibodies against pERK1/2 and anti-glutamine synthetase (GS) were used separately, not mixed in a cocktail.

**Table 1:** Details of primary antibodies used for IHC and WB

	Name	Product code	Host	Dilution	Company
IHC					
	Phospho-p44/42 MAPK (Erk1/2) (Thr202/Tyr204)	4376	Rabbit	1:50	Cell Signaling Technology, Danvers, MA, USA
	Anti IL-1 beta antibody	ab 9787	Rabbit	1:100	Abcam; Cambridge, UK
	Calcitonin-gene related peptide, polyclonal	B-47-1	Rabbit	1:800	Europroxima, Arnhem, Netherlands
	Phospho-p38 MAPK (Thr180/Tyr182)	9216	Mouse	1:200	Cell Signaling Technology, Danvers, MA, USA
	Phospho-SAPK/JNK (Thr183/Tyr185)	9252	Mouse	1:200	Cell Signaling Technology, Danvers, MA, USA
	Anti TNF-alpha antibody	ab66579	Rabbit	1:200	Abcam; Cambridge, UK
	Anti IL-6 antibody	ab6672	Rabbit	1:100	Abcam; Cambridge, UK
	Anti-Glutamine Synthetase Antibody, Clone GS-6	MAB302	Mouse	1:100	Merck Milipore, Darmstadt, Germany
WB					
	Phospho-p44/42 MAPK (Erk1/2) (Thr202/Tyr204)	4376	Rabbit	1:1000	Cell Signaling Technology, Danvers, MA, USA
	Anti IL-1 beta antibody	ab 9787	Rabbit	1:500	Abcam; Cambridge, UK
	Anti-Calcitonin Gene Related Peptide	C8198	Rabbit	1:250	Sigma-Aldrich, St. Louis, MO, USA
	Anti β-actin	Sc-477778	Mouse	1:5000	Santa Cruz Biotech, Santa Cruz, CA, USA
	GAPDH (D16H11) XP mAb	5174	Rabbit	1:1000	Cell Signaling Technology, Danvers, MA, USA

**Table 2:** Details of secondary antibodies used for IHC and WB

	Conjugate and host	Against	Dilution	Company
IHC				
	FITC (goat)	Anti-rabbit	1:100	Cayman Chemical, Ann Arbor, MI, USA
	Alexa 594 (goat)	Anti-mouse	1:100	Invitrogen, CA, USA
WB				
	HRP-conjugated	Anti-rabbit	1:2000	Cell Signaling Technology, Danvers, MA, USA
	HRP-conjugated	Anti-mouse	1:2000	Cell Signaling Technology, Danvers, MA, USA

### Microscopic analysis

Sections were examined and images were obtained by using a light- and epifluorescence microscope (Nikon 80i, Tokyo, Japan), coupled to a Nikon DS-2 MV camera. For image analysis FITC (480 nm), TRITC (540 nm) and DAPI (360 nm) filters were used. Adobe Photoshop CS3 (v.8.0; Adobe Systems, Mountain View, CA, USA) was used to visualize co-labelling by superimposing the digital images.

### Western blot

Animals for Western blot (12 for IS, 12 for CFA and 12 for vehicle) were perfused transcardially with PBS after 24 h or 7 days. Six fresh rats were used as controls. TG were immediately frozen on dry ice and stored at −80 °C until assay.

TG were homogenized in cell extract denaturing buffer (BioSource, Vacaville,CA, USA) containing phosphatase and protease inhibitor cocktails (Sigma, St. Louis, MO, USA). After centrifugation (12,000 rpm, 4 °C, 10 min) the supernatants were collected. Protein concentrations were measured with a protein assay reagent (Bio-Rad Laboratories, Hercules, CA, USA) and a Tecan Infinite M200 microplate reader. Protein samples were mixed with Laemmli Sample Buffer (Bio-Rad Laboratories, Hercules, CA, USA) and heated (95 °C, 4 min). Equal amounts (40 μg) of protein were loaded onto 4–15 % Ready Gel Precast Gels (Bio-Rad Laboratories, Hercules, CA, USA) with a molecular weight marker (Precision Plus Protein Standard, Bio-Rad Laboratories, Hercules, CA, USA). Gel electrophoresis was followed by blocking in 5 % non-fat milk or BSA and incubation with primary antibodies (see Table 1, 4 °C, overnight) and secondary antibodies (see Table 2, 1 h, room temperature). Finally, the membranes were visualized with Fujifilm LAS-1000 Luminescent Image Analyzer (Fujifilm, Stamford, CT, USA) and the band optical density ratio was quantified by using ImageJ software. Data were calculated relative to GAPDH as a loading control and further normalized to the fresh control (set to be 1) for each individual run.

### Myography

For myography studies, fresh rats (*n* = 5) were decapitated under CO_2_ anaesthesia. Middle meningeal artery (MMA) was dissected free, using dissection microscope, for details see Haanes and Edvinsson [20]. MMAs were isolated in 119 mM NaCl, 15 mM NaHCO_3_, 4.6 mM KCl, 1.2 mM MgCl_2_, 1.2 mM NaH_2_PO_4_, 5.5 mM glucose and 26 μM EDTA. After dissection, a buffer with similar composition, but including 1.5 mM CaCl_2_ and without EDTA was used. All solutions were aerated with gaseous mixture composed of 95 % O_2_ and 5 % CO_2_, to maintain a pH of 7.4.

Each ~2 mm long segment of the MMA (*n* = 5) from fresh rats was mounted in an arterial myograph, on a pair of 25 μm metal wires. One wire was connected to a micrometer screw where the vascular tone could be adjusted and the other to a force displacement transducer, paired with an analogue/digital converter (ADInstruments, Oxford, UK). Data were recorded on a computer through use of a PowerLab unit and LabChart (ADInstruments, Oxford, UK) was used for recording and calculations.

All experiments were conducted at 37 °C. The segments were normalized to 90 % of the internal circumference a vessel would have at 50 mmHg. A reference value for each segment was created by replacing part of the NaCl with KCl (60 mM K^+^); any segment with a maximum contractile capacity of less than 0.1 mm was excluded. Segments were stably precontracted with 30 mM K^+^. For both substances a cumulative concentration vs. response curve was made.

## Results

### HE staining

**Fig. 1:**
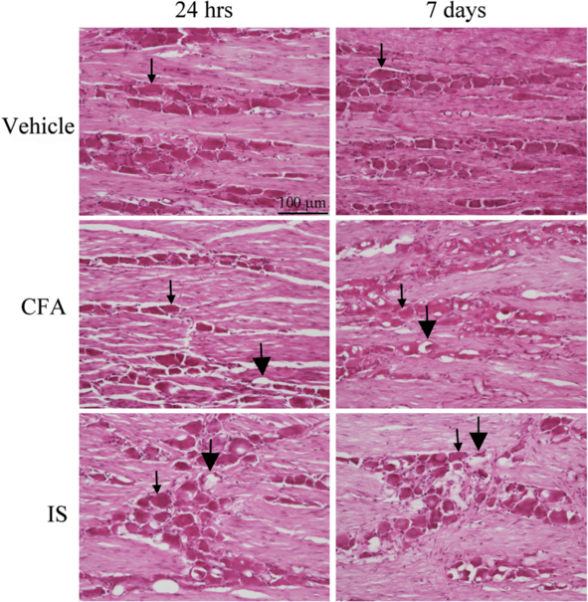
Hematoxylin-Eosin staining of TG from animals treated with vehicle (control), CFA and IS. The ganglia consists of bipolar neurons sorrounded by a single layer of SGCs (*thin arrows*). In the CFA and IS treated groups vacuoles (*thick arrows*) can be seen, as sign of tissue shinkrage and cell damage

HE staining of the TG is shown in Fig. 1. By keeping the orientation of the TG, we could identify the peripheral (cortical) and the central (medullary) zones, and also the V1, V2 and V3 regions of the TG. The staining revealed neurons of different sizes, enveloped by a single layer of SGCs. These neuron/SGC units were intermingled between fibers. The morphology of the different TGs was in general good, though tissue shrinkage was observed in some of the TGs.

### Immunohistochemistry

#### pERK1/2

**Fig. 2:**
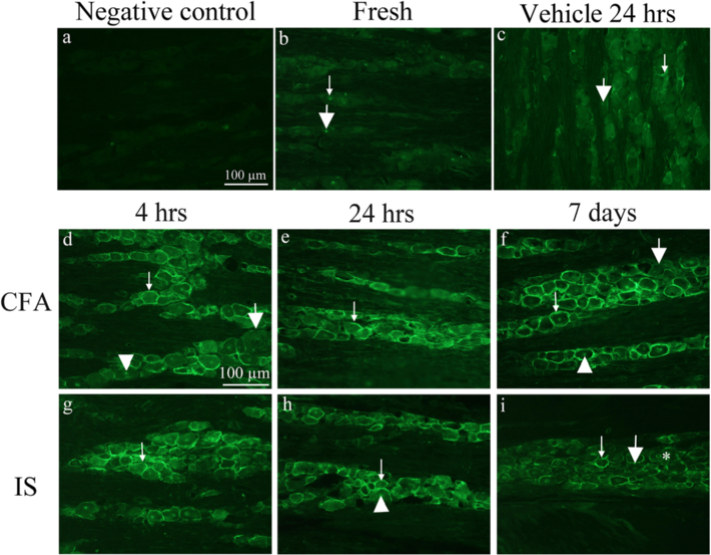
pERK immunohistochemistry on the TG. **a**. Negative control, showing no immunoreactivity. **b**. pERK1/2 staining in fresh animals. pERK positive nuclei (*thin arrow*) and nucleolei (*thick arrow*) can be detected. **c**. 24 h vehicle group shows weakly increased pERK1/2 immunoreactivity in the SGCs (*thin arrow*) and in the nucleolei (*thick arrow*). The TGs were studied at various time points (**d-i**). Positive immunoreactivity with different intensity can be detected in SGC (*thin arrows*) in all specimens. Negative SGC can also be seen (*thick arrows*). At 7 days CFA model, only few negative SGC were observed. Intensely stained nucleolei were also detected (*arrow heads*), more obvious at day 7 CFA. In the 7 days IS group, a lot of positive neuronal nuclei but no nucleolei were observed (*asterisk*)

pERK1/2 can be used as a dynamic, rapid and robust marker of noxious stimulation, as ERK1/2 phosphorylation can occur within a minute [21]. We were interested in the long term activation of pERK, as it could be related to permanent activation of the TG. pERK1/2 immunoreactivity was observed in a few nuclei and nucleoli of the neurons in the fresh TGs. No immunoreactivity was detected in the cytoplasm of the neurons or SGCs. In the 24 h vehicle group, some pERK1/2 immunoreactivity was found in the SGCs. The negative control, i.e. when the primary antibody was omitted, displayed no immunoreactivity (Fig. 2a-c).

In the CFA (Fig. 2d-f) and IS (Fig. 2g-i) treated rats, pERK1/2 immunoreactivity was observed in the SGCs at all three time points (4 h, 24 h and 7 days). At 4 h, only few SGC were activated in the V1 (anteromedial) region of the ganglion, compared to the peripheral zone (data not shown). No significant difference could be seen between the IS, CFA or the vehicle groups. At 24 h, high-intensity pERK1/2 immunoreactivity was detected in the anteromedial part of the TG in the SGCs in both IS and CFA specimens. A slight increase in pERK1/2 immunoreactivity was observed in the neuronal nucleoli in the 7 days specimens, in both the CFA and IS groups. No difference was seen between left and right side (data not shown).

**Fig. 3:**
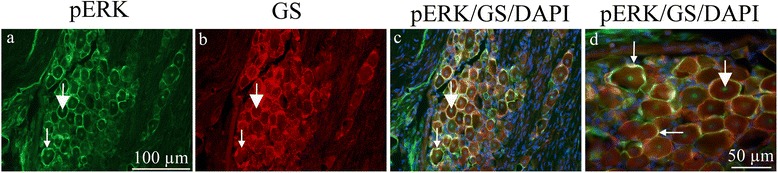
pERK1/2 and GS double immunohistochemistry on the TG 7 days after CFA. **a**. All SGCs seem to be immunoreactive. Thin arrow point at pERK1/2 immunoreactivity in SGCs, thick arrow at positive neuronal nucleolei. **b**. GS immunoreactivity was found in the SGCs. Thin arrow point at GS immunoreactivity. **c**. and **d**. Co-localization of pERK1/2 and GS is demonstrated in the merged images. Nuclei DAPI staining is included. Thin arrows point at pERK1/2/GS co-localization and thick arrows at positive neuronal nucleolei

To assess whether the pERK1/2 co-localized with the specific SGC marker GS, double immunohistochemistry was performed. Indeed, the markers co-localized (Fig. 3a-d), demonstrating that pERK1/2 is present in the SGCs.

## IL-1β

**Fig. 4:**
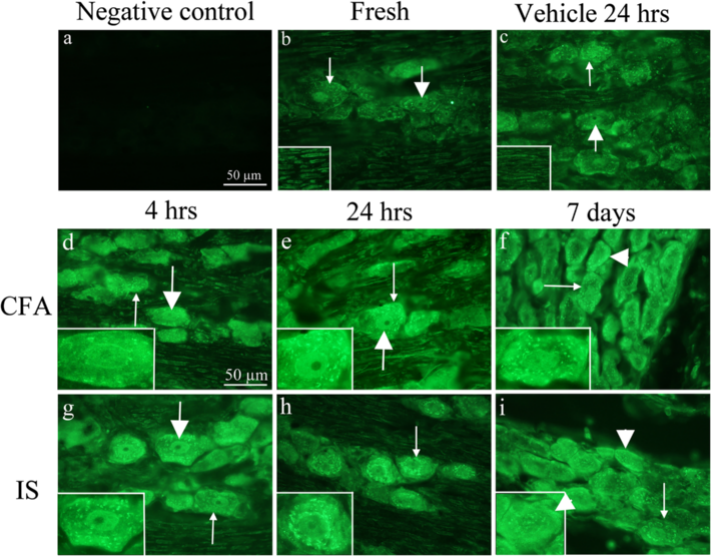
IL-1β immunohistochemistry on the TG **a**. Negative control showing no immunoreactivity. **b**. IL-1β staining in fresh animals. Immunoreactive neuronal nuclei could be detected (*thick arrow*). In addition, an intracytoplasmatic granular staining was seen (*thin arrow*). **c**. 24 h vehicle group showed a similar pattern as for fresh animals. Inserts immunoreactive nerve fibers. **d-i**. Immunoreactivity was detected in the neuronal nuclei, but no nucleolei in the CFA and IS treated groups (*thick arrows*, **d-i**). In the cytoplasm immunoreactivity was detected in a granular manner (*thin arrows*, **d-i**). In the 7 days CFA and IS groups, increased immunoreactivity was seen as a condensed, homogeneous material close to the cell membrane (**i**, *arrow heads*)

IL-1β plays a pivotal role in inflammation and has been shown to be present in the spinal cord, following the injection of CFA into the rat hind paw [22]. We were therefore interested if similar changes could occur in the TG after inflammatory activation. The negative control displayed no IL-1β immunoreactivity (Fig. 4a). In fresh TG, IL-1β immunoreactivity was observed in the neuronal cytoplasm (in a granular manner), in a few nuclei and in the nerve fibres (Fig. 4b). The same pattern was found in the vehicle-treated animals (Fig. 4c).

The most interesting finding is the activation pattern of IL-1β. Firstly we do not see any activation in the vehicle control compared to fresh, which illustrates that this is a pure effect of the inflammatory substances added on the dura. In the CFA (Fig. 4d-f) and IS (Fig. 4g-i) treated rats, IL-1β immunoreactivity was observed intracellularly in the neurons and in the fibres at all three time points. However, we do see a difference in the pattern of staining at the different time points. At 4 h there is more of the granular staining in the IS compared to the CFA, suggesting a fast response/activation from the IS. Similar increase in the granular pattern is observed after 24 h post the CFA stimulation, at this time point there is no big difference between CFA and IS. At day seven a “ring” of IL-1β immunoreactivity below or close to the neuronal cell membrane was evident, which differed from the granular pattern observed in the fresh TGs (Fig. 4b). This is clearly stronger in the CFA compared to the IS treated rats. No immunoreactivity was observed in the SGCs. No difference was detected between the left and right sides.

## CGRP

Previous experiments have revealed a strong relationship between the release of IL-1β and CGRP in the neurons. Pre-exposure of trigeminal neurons to an IL-1β conditioned medium led to an increased CGRP release in the neurons after stimulation with capsaicin. CGRP is also the main peptide believed to be involved in migraine pathophysiology.

**Fig. 5:**
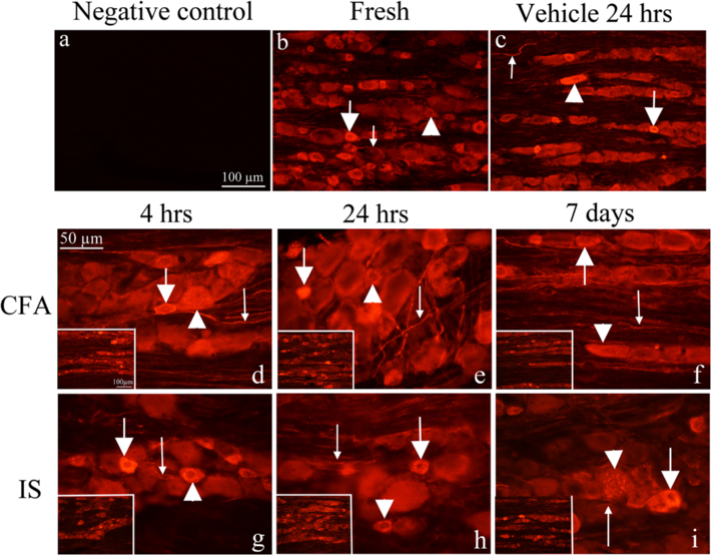
CGRP immunohistochemistry on the TG. **a**. No immunoreactivity was observed in negative controls. **b**. In fresh animals pearl like staining was observed in thin fibres (*thin arrow*). Homogeneous staining was detected in small neurons (*thick arrow*) as well as intracytoplasmatic granular staining (*arrow head*). **c**. A similar immunoreactivity as for fresh animals was detected in the vehicle group, with thin fibre (*thin arrow*), small neurons (*thick arrow*) and granular cytoplasmatic (*arrow head*) staining. **d-i**. A pearl like staining was observed in thin fibres (*thin arrow*), homogeneous staining was detected in the small neurons (*thick arrow*) as well as intracytoplasmatic granular staining (*arrow head*). No difference could be found between the different algesics and time points. Inserts, smaller magnification

Our negative control did not exhibit immunoreactivity (Fig. 5a). In fresh TG, neurons and nerve fibers were CGRP immunoreactive (Fig. 5b). The immunoreactivity was observed in the neuronal cytoplasm. The staining ranged between homogeneous staining of the cytoplasm to a granular immunoreactive pattern around the nucleus, possibly in the ER. A limited number of thin, CGRP-positive pearl-like fibers were also observed in the TG. No immunoreactivity was seen in the SGCs. The same pattern as for the fresh TG was seen in the vehicle-treated animals (Fig. 5c).

In the CFA (Fig. 5d-f) and IS (Fig. 5g-i) treated rats, CGRP immunoreactivity was observed in the neurons and nerve fibres at all three time points. As compared with the control specimens (fresh and vehicle-treated rats, Fig. 5b-c), we observed an increase in immunoreactive fibres (Fig. 5d-i), particularly in the CFA treated animals, this increased staining of the fibres is persistent at all time-points (Fig. 5d-i).

## Other antibodies tested

We additionally studied the immunoreactivity of TNF-α, IL-6, pp38 and pJNK, but in our hands these proved negative (data not shown).

### Western blot

**Fig. 6:**
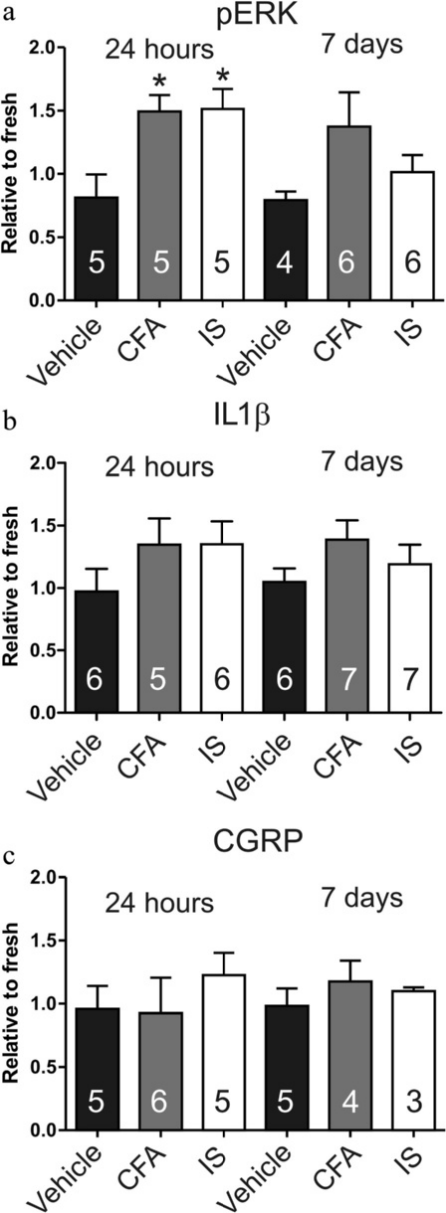
Average data from Western blots of the TG. **a** Western blot for pERK. The level of pERK is higher in CFA and IS treated groups. *, *P* < 0.05, one-way ANOVA, with Bonferroni post-test. **b** Western blot for IL-1β. There is a tendency of increased IL-Iβ activation following CFA or IS application on the dura. No significant difference was seen between the groups. **c** Western blot for CGRP. No significant changes in CGRP activation following CFA or IS application on the dura

Since immunohistochemistry is generally better for observing “ON/OFF” changes in expression together with protein localization, we aimed to more quantitatively investigate the protein expression in the TG. Western blots therefore performed for pERK1/2, IL-1β and CGRP (representative blot can be found in Additional file 1: Figure S1). The only significant increase was seen for pERK1/2 after stimulation with both CFA and IS for 24 h (Fig. 6a). We did not see significance IL-1β or CGRP (Fig. 6b-c). We did, however, see a tendency for an increase in IL-1β, particularly after 24 h of stimulation with CFA or IS. Significance was not reached, which could be due to the complete ganglion was used for Western blot. However, we do observe the same tendencies as for our immunostainings.

### Myography

**Fig. 7:**
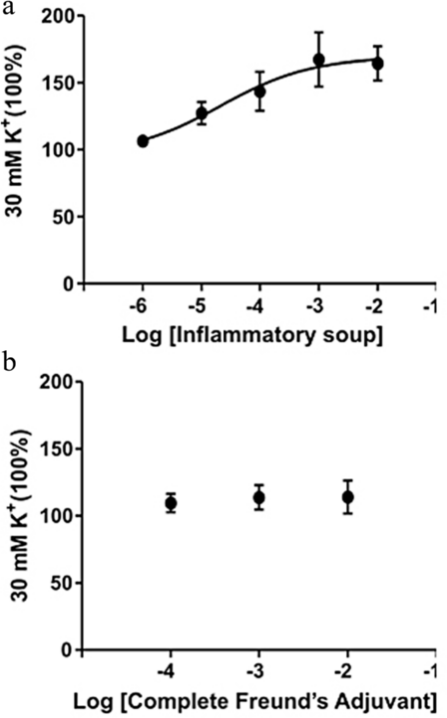
Myograph recordings on middle menigeal arteries. **a** IS caused a vasocontraction of the middle menigeal artery. **b** CFA did not show any significant changes in vasocontractility (*n* = 5)

In our model, we observe activation of the trigeminal ganglion using the inflammatory substances CFA and IS. Based on the vascular theory of migraine, we wanted to see if the agents caused vasodilation or vasocontraction when applied to the middle meningeal artery. MMAs from fresh rats were isolated and mounted in a wire myograph. IS caused a strong contraction of the MMAs (Fig. 7a), whereas CFA did not result in any vasomotor response (Fig. 7b). It is worth mentioning that CFA consists largely of mineral oil and is not water soluble. No vasomotor effects occurred when CFA was added directly onto the MMA in the myograph (data not shown).

## Discussion

To the best of our knowledge the present study is the first study to examine the inflammatory response in the TG after the application of algesic substances onto the dural receptive field. The local effects of inflammatory agents on the MMA, and neuro-glial activation in the TG, were followed.

The vasogenic theory has recently been questioned in consequence of the lack of evidence of major cranial vasodilatation during migraine attacks [23, 24]. It is currently conceived that the activation of the trigeminovascular system after chemical stimulation of the dura is explained by activation of perivascular sensory fibres, leading to hypersensitivity to mechanical and thermal stimuli [8, 9]. Previous studies of the onset of this hypersensitivity [8] have indicated that peripheral sensitization occurs rapidly, within 20 min after the application of inflammatory agents onto the dural surface and central sensitization becomes established after 120–240 min [11]. Based on these previous findings we speculated that hypersensitivity and trigeminovascular activation could be maintained for a longer period of time (7 days) and that application of algesic substances onto the dura mater could be used as an animal model for long term activation of the trigeminovascular system.

The inflammatory soup consisted of bradykinin, histamine, serotonin and prostaglandin E2, which are found to be released endogenously during inflammation and have a role in neurogenic inflammation [4, 8, 9, 25]. CFA is considered to be an important immune-potentiator which is widely used in peripheral pain models [26, 27]. Interestingly, we see a similar pattern between the right and left TG, even though the inflammatory agents were added on only one side. This could be explained by earlier tracing studies that characterize the innervation of the dura mater where the application of the labelling substances on a caudomedial region of the dura mater, labelled neurons appeared bilaterally [28]. In fresh ganglia, only minor pERK1/2 immunoreactivity was seen (Fig. 2). The immunoreactivity increased at the early time points and was extended up to 7 days. There was no significant difference between the CFA and IS groups. We observed a pERK1/2 immunoreactivity in the vehicle treated SGCs as compared to fresh TG. This might be explained by the surgical procedure itself. There are numerous extracranial pain-sensitive structures including skin, muscles and periosteum [29, 30]. Furthermore, it should be taken into consideration that nociceptive fibres also undergo activation during removal of skull bones [13]. Previous studies have indicated a decrease in pERK1/2 activation, with return to basal levels within 2 h after stimulation [13, 31, 32]. In our experimental setup, increased pERK1/2 immunoreactivity was detected at both 24 h and 7 days after stimulation. This might suggest that pERK1/2 activation depends on the stimulated dural area and the exposure time of the inflammatory agents.

Prolonged activation of pERK1/2, participates in the regulation of nuclear proteins and transcription factors, and might be a critical determinant in sustained activation of V1 region in the TG. Our findings suggest an interplay between neurons and SGCs, were SGCs could serve an role in the pathophysiology of inflammation and pain. After the activation of the dural-sensitive trigeminal neurons, it has previously been shown that the neurons release CGRP which stimulates the SGCs to release pro-inflammatory cytokines which can further stimulate the TG neurons [31, 33].

In a previous study involving a transfected rat sciatic nerve model, the upregulation of IL-1β was found 35 days after surgery, suggesting that it may have a role in maintained neuropathic pain [34]. In our results we show an increase in the IL-1β as early as 4 h for the IS and at 24 h for both IS and CFA. Interestingly, at 7 days, we observed a dense staining close to the cell membrane. We cannot fully explain this staining, but it might suggest a higher packing density of IL-1β close to the cell membrane which could indicate a potentially increased release. Importantly, earlier studies have detected IL-1β receptors on the surface of SGCs [35].

Following stimulation with IL-1β, SGCs have been shown to be activated, which in turn may influence the neuronal responses [36]. Previous cell culture studies have demonstrated an elevated release of CGRP while incubation with IL-1β [36, 37], mediated by the MAPK activation [38–40]. Our findings of increased levels of pERK1/2, CGRP fibres and IL-1β support the view of a possible interaction between these three molecules after induced local inflammation. A key role has recently been ascribed to increased CGRP release in the TG neurons [41] and CGRP receptor antagonists have a therapeutic effect in migraine and other pain syndromes [27, 42] that could to act on the TG [43]. Indeed, also in this inflammation activated TG, we observe an increase in the CGRP after 7 days.

Our Western blot studies revealed the same tendency with significantly elevated level of pERK1/2 and a tendency of elevated IL-1β and CGRP after CFA or IS but where the differences were statistically not significant. It is worth pointing out that for the immunohistochemistry, detailed microscopy, allowed us to focus on the V1 region of the TG, whereas in the Western blot, the protein measurements were made on whole TG as it is impossible macroscopically to localize the exact area to analyse. In addition, the TG for Western blot is not fixed with perfusion, thus some trigeminal activation can occur during the tissue dissection.

Based on the vascular theory of migraine, we wanted to see if the agents caused vasodilation or vasocontraction when applied to the middle meningeal artery. Interestingly, the IS caused a strong contraction of the artery, whereas nothing occurred with the artery tone following the CFA administration. Vasodilatation has been suggested to be the initiator of migraine, but could also be a consequence or a side phenomenon [44] and it has previoulsy been shown that not all migraine attacks are associated with vasodilation [45]. An earlier study supporting the vascular hypothesis found that a short period of vasoconstriction triggers the release of vasodilator agents, such as CGRP, and could explain some of the findings presented here [42]. Overall, we show that the contractile IS and non-vasoactive CFA causes activation of the TG suggesting that vasodilation of the MMA is not the trigger the TG activation.

## Conclusion

The present study has demonstrated that the application of IS or CFA onto the dural surface of rats can be used as a method to induce changes in the expression of pERK1/2, IL-1β and CGRP positive nerve fibres in the TG. These findings suggest a possible immuno-neuro-glial interaction. This opens up an interesting line for further investigations on the central effects of inflammatory agents and their relation to migraine and pain.

## Additional file

Additional file 1: Figure S1. Representative Western blot. The figure shows a representativce Western blot for the data that are presented in Fig. 6. (TIFF 2843 kb)
