# Supplementary figures and images for: Dural administration of inflammatory soup or Complete Freund’s Adjuvant induces activation and inflammatory response in the rat trigeminal ganglion
Source: J Headache Pain. 2015 Sep 2;16:79. doi: 10.1186/s10194-015-0564-y (PMC4556720; doi:10.1186/s10194-015-0564-y)

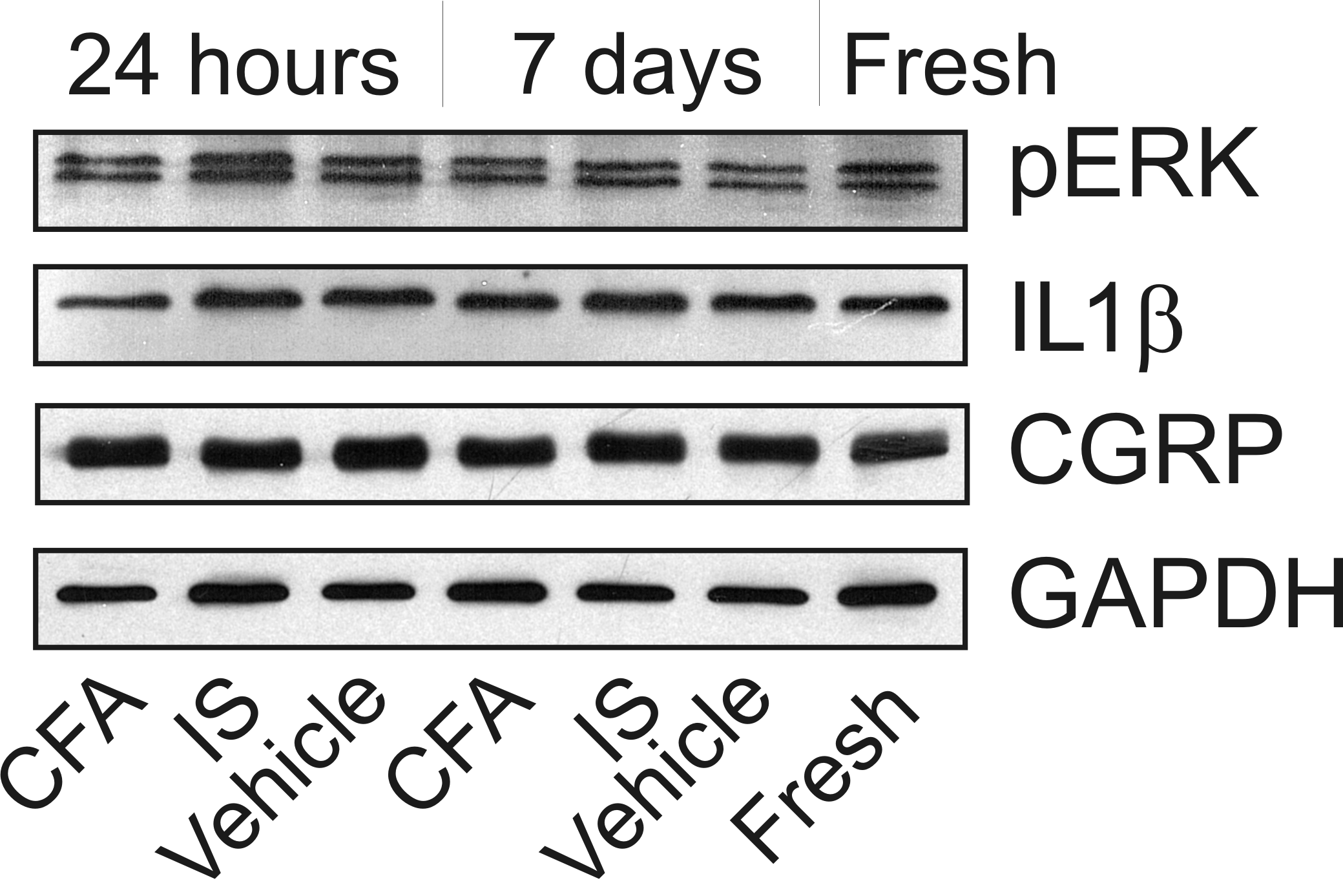

Supplement: Additional file 1: Figure S1. — Representative Western blot. The figure shows a representativce Western blot for the data that are presented in Fig. 6. (TIFF 2843 kb) [file 10194_2015_564_MOESM1_ESM.tif]
